# Risk for rabies transmission from encounters with bats, Colorado, 1977-1996.

**DOI:** 10.3201/eid0503.990315

**Published:** 1999

**Authors:** W J Pape, T D Fitzsimmons, R E Hoffman

**Affiliations:** Colorado Department of Public Health and Environment, Denver, USA.

## Abstract

To assess the risk for rabies transmission to humans by bats, we analyzed the prevalence of rabies in bats that encountered humans from 1977 to 1996 and characterized the bat-human encounters. Rabies was diagnosed in 685 (15%) of 4,470 bats tested. The prevalence of rabies in bats that bit humans was 2.1 times higher than in bats that did not bite humans. At least a third of the encounters were preventable.


### Risk for Rabies Transmission from Encounters with Bats, Colorado, 1977–1996

**W. John Pape,* Thomas D. Fitzsimmons,*† and Richard E. Hoffman***
*Colorado Department of Public Health and Environment, Denver, Colorado, USA; and †Centers for Disease Control and Prevention, Atlanta, Georgia, USA

To assess the risk for rabies transmission to humans by bats, we analyzed the prevalence of rabies in bats that encountered humans from 1977 to 1996 and characterized the bat-human encounters. Rabies was diagnosed in 685 (15%) of 4,470 bats tested. The prevalence of rabies in bats that bit humans was 2.1 times higher than in bats that did not bite humans. At least a third of the encounters were preventable.

Although no cases of human rabies have been reported since 1931 in Colorado, rabies remains a health risk in this state because of the frequency with which Coloradans have contact with bats. The first objective of this study was to determine the prevalence of rabies in bats that were submitted for laboratory testing in Colorado over a 20-year period, including an analysis by bat species. The second objective was to characterize the circumstances of confirmed bat-human encounters during this same period and to evaluate how this information could be used to prevent human rabies.

### Data Sources

### Laboratory Records

Rabies diagnosis was conducted by two laboratories in Colorado: the Colorado Department of Public Health and Environment (CDPHE) Laboratory and the Colorado State University (CSU) Veterinary Diagnostic Laboratory. Bats were accepted for testing from public and private sources if they had had contact with a person or a domestic pet, if the possibility of contact could not be excluded, or if the bat exhibited abnormal behavior. County agencies were also permitted to submit up to three bats per week (usually found dead from no apparent cause or exhibiting aberrant behavior) for local surveillance. None of the submissions were for studies of rabies prevalence among bats with a normal appearance or in their natural habitat.

Records from both laboratories were maintained by the CDPHE Epidemiology Division and made up the first dataset we analyzed. Information extracted from these records included rabies test date, test result, and bat bite information. For bats sent to CDPHE (but not CSU), laboratory technicians identified the bats by species, and the data were included in the analysis.

### Possible Rabies Exposure Memoranda

A second dataset consisted of memoranda describing any animal exposure reported to CDPHE resulting in rabies postexposure prophylaxis (PEP). Memoranda were not written for encounters in which the animal tested negative for rabies, even if a person was bitten. Infrequently, CDPHE staff wrote memoranda before learning that an animal had tested negative for rabies or when PEP was recommended but not administered. Four persons in the Epidemiology Division worked on zoonosis control during the 20-year study period; two of them wrote 90% of the memoranda. A bat encounter was defined as bat contact or possibility of bat contact with a person. A wound was defined as a visible puncture, scratch, bleeding, or a sensation of sharp pain during the encounter. No attempt was made to distinguish bite wounds from claw marks or scratches.

The analysis of circumstances was restricted to memoranda in which the presence of a bat was

Address for correspondence: W. John Pape, 4300 Cherry Creek S, Denver, CO 80246, USA; fax: 303-782-0904; e-mail: john.pape@state.co.us.

documented. Memoranda that described encounters with bats were identified, and data on person, place, and time were extracted. The circumstance of encounter was listed as one of 13 general categories that best described the event. Any person who initiated the standard rabies PEP series was designated as having received treatment.

### Findings

### Laboratory Records

From 1977 through 1996, 4,502 bats were submitted for testing. CDPHE received 4,394 bats (98%), and CSU received 108 bats (2%). These bats represented 15 (83%) of 18 species present in Colorado (1) (Table 1). Thirty-two bats were excluded from further analysis because either the test result or the bite status was unrecorded. Rabies was diagnosed in 685 (15%) bats and accounted for 98% of all animal rabies cases in Colorado during the study period. Of the 233 bats that bit people, 69 (30%) had rabies. Of the 4,237 bats that did not bite people, 613 (14%) had rabies. The prevalence of rabies among bats that bit humans was 2.1 times higher (95% confidence interval 1.7 to 2.5) than in bats not involved in human bites. None of the persons bitten by bats got rabies. Human rabies has not been reported in Colorado since 1931 (CDPHE, unpub. data, 1998).

Species data were available for 4,182 (94%) bats. Three species—big brown bats (*Eptesicus fuscus*), hoary bats (*Lasiurus cinereus*), and silver-haired bats (*Lasionycteris noctivagans*)—accounted for 73% of total submissions, 84% of the rabies-positive specimens, and 70% of bats involved in bite incidents. The prevalence of rabies in silver-haired bats (5%) was lower than in big brown bats (17%) or hoary bats (40%).

Table 1. Prevalence of rabies in bats submitted for testing, Colorado, 1977–1996

| Species | No. that bit humans (% rabid) | No. that did not bite humans (% rabid) | Total no. tested (% rabid) |
|---|---|---|---|
| Big brown bat *Eptesicus fuscus* | 122 (27) | 2,013 (16) | 2,135 (17) |
| *Myotis* genus group[a] | 35 (14) | 722 (6) | 757 (7) |
| Silver-haired bat *Lasionycteris noctivagans* | 28 (14) | 628 (5) | 656 (5) |
| Hoary bat *Lasiurus cinereus* | 13 (77) | 452 (39) | 465 (40) |
| Long-eared bat *Myotis evotis* | 8 (88) | 38 (21) | 46 (33) |
| Brazilian free-tailed bat *Tadarida brasiliensis* | 0 (0) | 41 (12) | 41 (12) |
| Red bat *Lasiurus borealis* | 1 (100) | 25 (8) | 26 (12) |
| Pallid bat *Antrozous pallidus* | 0 (0) | 21 (5) | 21 (5) |
| Big free-tailed bat *Nyctinomops macrotis* | 2 (50) | 19 (11) | 21 (14) |
| Townsend's big-eared bat *Plecotus townsendii* | 1 (0) | 13 (0) | 14 (0) |
| Species data unavailable | 23 (35) | 265 (9) | 288 (11) |
| Total | 233 (30) | 4,237 (14) | 4,470 (15) |

[a]Includes six species in the genus *Myotis* that could not be easily distinguished by inspection: *M. lucifugus, M. volans, M. thysanodes, M. californicus, M. ciliolabrum,* and *M. yumanesis.*

### Memoranda

During the 20-year study period, 271 memoranda described possible encounters with bats; 240 (89%) memoranda documented the presence of a bat and were included in the analysis. Of the 131 bats tested, 99 had rabies.

Of the 240 persons who encountered bats, 141 (59%) were male and 99 (41%) were female. From the 195 (81%) records that recorded the person's age, the range in age was 10 months to 81 years, and the median age was 25 years. Of the 182 (76%) persons reporting that they were wounded, the most common wound site was the hand (59%), followed by the arm (14%), head/neck (12%), leg/foot (9%), torso (2%), or multiple sites (2%).

Not enough information was available to characterize the time of day of the encounters. Two hundred (83%) encounters occurred between June and September, corresponding to peak activity periods and seasonal migratory patterns of bats in Colorado.

In the 217 records that noted location of bat encounters, 117 (54%) occurred outdoors, chiefly on home properties and park and recreation areas (none were reported in caves). Of the 100 (46%) bat encounters inside buildings, 83 were in private homes (37 of these in bedrooms). Big brown bats, colonial bats that commonly roost inhouses and buildings, were encountered more frequently outside than inside (60% vs. 39%) when rabid (n = 46). All 11 rabid hoary bats, solitary tree dwellers, were encountered outdoors. However, rabid silver-haired bats, another solitary tree-roosting species, were encountered equally indoors (n = 3) and outdoors (n = 3).

The four most frequent circumstances in which people encountered bats, accounting for 62% of the encounters, were a bat landing on an awake person (19%), a person picking up a grounded bat outside (18%), a person awakening to find a bat in the room (15%), and a person trying to remove a bat from inside a structure (10%). The remaining nine circumstances occurred repeatedly but less frequently (Table 2).

Of the 240 persons who had encounters with bats, 216 (90%) initiated PEP; nine of these stopped treatment after the bat tested negative for rabies. The bat tested negative in 17 of the 24 cases in which PEP was not administered, but a memorandum was written before the test results were available. The remaining seven persons did not receive prophylaxis because they or their physician did not believe that the contact warranted treatment. In three of these seven encounters (which occurred before 1983), the bat was found to be rabid, but no definite wound was observed.

The time from bat encounter to initiation of treatment could be calculated for 199 (92%) of the 216 patients who received PEP and was 1 hour to 28 days. Fifty percent of patients received their first dose of vaccine within 24 hours of exposure; 75% started treatment within 72 hours. Of the 18 patients who initiated treatment 7 or more days after the encounter, nine did not do so until advised by an acquaintance or physician of the possible rabies risk.

Table 2. Circumstances in which humans encountered bats, Colorado, 1977–1996

| Circumstances | Bat captured and tested: Rabid | Bat captured and tested: Not rabid | Bat not tested | All encoun-ters |
|---|---|---|---|---|
| Bat landed on person | 17 | 2 | 27 | 46 |
| Person picked up bat outdoors | 24 | 5 | 15 | 44 |
| Person awoke to find bat in room | 17 | 4 | 14 | 35 |
| Person tried to remove bat from indoors | 5 | 2 | 17 | 24 |
| Person inadvertently touched hidden bat | 3 | 5 | 8 | 16 |
| Person handled captured bat | 12 | 0 | 1 | 13 |
| Child found alone with bat | 4 | 3 | 2 | 9 |
| Person handled bat as part of job | 6 | 1 | 1 | 8 |
| Person stepped on bat | 3 | 0 | 3 | 6 |
| Person bitten while taking bat from pet | 2 | 1 | 3 | 6 |
| Person bitten by pet that had bat in mouth | 1 | 4 | 1 | 6 |
| Person attributed wound to bat they saw | 0 | 2 | 0 | 2 |
| Other circumstances | 0 | 0 | 6 | 6 |
| Unspecified in report | 5 | 3 | 11 | 19 |
| Total | 99 | 32 | 109 | 240 |

### Silver-Haired Bats

Although the silver-haired bat rabies virus variant was isolated from 15 of the 21 persons who died of bat-associated rabies in the United States from 1980 through 1997, we observed that silver-haired bats in Colorado had neither the greatest frequency nor the highest species-specific rate of rabies. Our findings are consistent with tabulations from New York (1988 to 1992) and Arkansas, Virginia, and West Virginia (1990 to 1994), which showed that silver-haired bats made up a small proportion of bats submitted for rabies testing; only a small number of submitted silver-haired bats were rabies positive (2,3). Nonetheless, Arkansas (1991), New York (1993), and West Virginia (1994) each had human cases associated with the rabies virus variant common to silver-haired bats (4-6). Because the frequency of human encounters with this species is apparently low and the prevalence of rabies in tested silver-haired bats is small, other factors must explain the silver-haired bats' association with human rabies cases. One hypothesis is that silver-haired bats are more aggressive than other bats (1). Additionally, one study has demonstrated that the rabies virus variant of silver-haired bats replicates in nonneuronal tissue more efficiently than a coyote rabies virus variant (7). This attribute might explain how a small dermal inoculum of silver-haired variant rabies virus from a seemingly superficial bite could cause infection. As silver-haired and hoary bats are tree dwellers that favor old growth forest habitat, it should be unexpected to encounter them indoors. None of the 12 hoary bats (11

rabid) included in this series were encountered inside. In contrast, three of the nine encounters with silver-haired bats were indoors. All three bats were rabid.

### Conclusions

Bats that interact with humans are far more likely to have rabies than bats that avoid humans, and rabies prevalence is highest in bats that bite. Conversely, rabid bats appear to interact more frequently and to be more prone to bite than nonrabid bats. This behavior is consistent with clinical manifestations of rabies in wildlife species in which the animal exhibits abnormal behavior, loses its natural fear of humans, and acts aggressively (8).

Encounters with bats resulted from a relatively small number of recurring situations. At least a third of the encounters (picking up grounded bats, handling captured bats, and trying to remove bats from structures or from pets' mouths) were preventable; however, most encounters in which a person inadvertently touched a hidden bat or a bat landed on a person were probably unavoidable.

Delays in treatment suggest that some people may not be aware of the risk for rabies transmission from contact with a bat. In the United States, in nearly half of the cases of human rabies associated with bat variant rabies virus, the person had no history of contact with a bat (9-11). Although unrecognized exposures may have occurred, the persons involved probably did not understand the risk after exposure to a bat and therefore did not seek medical care. Even when specifically asked about animal exposures, some patients and their families initially did not report bat contact (11,12).

This study has several potential limitations. First, we do not know whether the prevalence of rabies in tested bats is representative of all bats that encounter humans. The laboratory testing was a passive surveillance system, dependent on the submission of bats by persons involved in encounters. We calculated a lower limit estimate of the prevalence of rabies among bats that bit humans by using data from the memoranda. If one assumed that the bats that bit humans and later escaped and thus were not tested (90) were rabies-free, the prevalence of rabies among bats that bit people would decrease from 30% (69 of 233) to 21% (69 of 323), still significantly higher than the prevalence in bats that did not bite people ($p < .001$).

The circumstances were categorized for a small proportion of all bat encounters. Memoranda were written for only 131 of 4,502 bats submitted for testing and for 109 encounters in which the bat escaped. The number of unreported encounters cannot be estimated, and information from those encounters could alter the frequencies of the type of encounter presented in this study.

Finally, administration of rabies PEP is not reportable in Colorado and, therefore, the study may not have included all persons who received PEP after a bat encounter. Because the state health department was the primary source of rabies biological supplies for medical providers in the state from 1977 through 1985, nearly all exposures requiring PEP would have come to the department's attention. Rabies biological supplies were more widely available from other sources after 1985. Although the number of reported PEP administrations remained stable, some exposures may not have been reported.

Two findings of this study support recent revisions of the Advisory Committee on Immunization Practices (ACIP) recommendations (13-15): bats that encountered humans had a high prevalence of rabies, and the third most frequently reported circumstance was a person awakening to find a bat in the room. The ACIP stated in October 1997 that PEP may be appropriate even in the absence of demonstrable bite, scratch, or mucous membrane exposures in situations in which such exposure is likely to have occurred (e.g., a sleeping person awakes to find a bat in the room or an adult finds a bat in a room with an unattended child, a mentally deficient person, or an intoxicated person) (14). Of 35 instances reported in this study in which a bat was found in the room by a person upon awakening, 17 bats were rabid, and 23 persons had evidence of a bite.

The Colorado Department of Public Health and Environment, the Colorado Division of Wildlife, and the Colorado Bat Society recently collaborated to publish an educational pamphlet that describes methods to prevent rabies exposure from a bat and measures to take if a person encounters a bat (16).

### Acknowledgments

We thank rabies laboratory workers for invaluable service over the past 20 years, especially Larry Briggs and Jane Carman. We also thank John Emerson for his diligence in investigating and documenting bat encounters from 1977 to 1986.

John Pape is an epidemiologist with the Communicable Disease Program, Colorado Department of Public Health and Environment. He has statewide responsibility for zoonotic disease surveillance and control. In addition to bat rabies, his research focuses on the epidemiology of zoonotic diseases in the western United States, including plague, hantavirus, and tick-borne relapsing fever.

### References

1. Armstrong DM, Adams RA, Navo KW, Freeman J, Bissell SJ. Bats of Colorado: shadows in the night. 2nd ed. Denver (CO): Colorado Division of Wildlife; 1996. p. 11.
2. Childs JE, Trimarchi CV, Krebs JW. The epidemiology of bat rabies in New York state, 1988-92. Epidemiol Infect 1994;113:501-11.
3. Dreesen DW, Orciari LA, Rupprecht CE. The epidemiology of bat rabies in the southeastern United States 1990-1994 [abstract]. In: Proceedings from 7th Annual International Meeting of Advances Towards Rabies Control in the Americas; 1996 Dec 9-13; Atlanta, Georgia. p. 44.
4. Centers for Disease Control. Human rabies—Texas, Arkansas, and Georgia, 1991. MMWR Morb Mortal Wkly Rep 1991;40:765-9.
5. Centers for Disease Control and Prevention. Human rabies—New York, 1993. MMWR Morb Mortal Wkly Rep 1993;42:799,805-6.
6. Centers for Disease Control and Prevention. Human rabies—West Virginia, 1994. MMWR Morb Mortal Wkly Rep 1995;44:86-7,93.
7. Morimoto K, Patel M, Corisdeo S, Hooper DC, Fu ZF, Rupprecht CE, et al. Characterization of a unique variant of bat rabies responsible for newly emerging human cases in North America. Proc Natl Acad Sci U S A 1996;93:5653-8.
8. Kaplan C, Turner GS, Warrell DA. Rabies: the facts. 2nd ed. Oxford: Oxford University Press; 1986. p. 72-4.
9. Centers for Disease Control and Prevention. Human rabies—Montana and Washington, 1997. MMWR Morb Mortal Wkly Rep 1997;46:770-4.
10. Centers for Disease Control and Prevention. Human rabies—Texas and New Jersey, 1997. MMWR Morb Mortal Wkly Rep 1997;47:1-5.
11. Centers for Disease Control. Human rabies—Texas, 1990. MMWR Morb Mortal Wkly Rep 1991;40:132-3.
12. Centers for Disease Control and Prevention. Human rabies—Connecticut, 1995. MMWR Morb Mortal Wkly Rep 1996;45:207-9.
13. Advisory Committee on Immunization Practices. Revised ACIP rabies post-exposure prophylaxis (PEP) statement—1997 Oct 22.
14. Constantine DG. Bat rabies in the southwestern United States. Public Health Rep 1967;82:867-8.
15. Rabies prevention—United States, 1991: recommendations of the Immunization Practices Advisory Committee (ACIP). MMWR Morb Mortal Wkly Rep 1991;40:1-19.
16. Colorado Division of Wildlife, Colorado Department of Public Health and Environment, Colorado Bat Society. Bats and rabies [pamphlet]. Denver (CO): The Department; 1997.

